# Characteristics of photosynthesis and vertical canopy architecture of citrus trees under two labor-saving cultivation modes using unmanned aerial vehicle (UAV)-based LiDAR data in citrus orchards

**DOI:** 10.1093/hr/uhad018

**Published:** 2023-02-08

**Authors:** Yuanyong Dian, Xiaoyang Liu, Lei Hu, Jinzhi Zhang, Chungen Hu, Yongzhong Liu, Jinxin Zhang, Wenbo Zhang, Qingqing Hu, Yahao Zhang, Yanni Fang, Jingjing Zhou

**Affiliations:** College of Horticulture & Forestry Sciences, Huazhong Agricultural University, Wuhan 430070, China; Hubei Engineering Technology Research Center for Forestry Information, Wuhan 430070, China; College of Horticulture & Forestry Sciences, Huazhong Agricultural University, Wuhan 430070, China; Hubei Engineering Technology Research Center for Forestry Information, Wuhan 430070, China; College of Horticulture & Forestry Sciences, Huazhong Agricultural University, Wuhan 430070, China; Hubei Engineering Technology Research Center for Forestry Information, Wuhan 430070, China; College of Horticulture & Forestry Sciences, Huazhong Agricultural University, Wuhan 430070, China; National Key Laboratory for Germplasm Innovation & Utilization of Horticultural Crops, Wuhan 430070, China; College of Horticulture & Forestry Sciences, Huazhong Agricultural University, Wuhan 430070, China; National Key Laboratory for Germplasm Innovation & Utilization of Horticultural Crops, Wuhan 430070, China; College of Horticulture & Forestry Sciences, Huazhong Agricultural University, Wuhan 430070, China; National Key Laboratory for Germplasm Innovation & Utilization of Horticultural Crops, Wuhan 430070, China; College of Horticulture & Forestry Sciences, Huazhong Agricultural University, Wuhan 430070, China; National Key Laboratory for Germplasm Innovation & Utilization of Horticultural Crops, Wuhan 430070, China; College of Horticulture & Forestry Sciences, Huazhong Agricultural University, Wuhan 430070, China; National Key Laboratory for Germplasm Innovation & Utilization of Horticultural Crops, Wuhan 430070, China; College of Horticulture & Forestry Sciences, Huazhong Agricultural University, Wuhan 430070, China; Hubei Engineering Technology Research Center for Forestry Information, Wuhan 430070, China; College of Horticulture & Forestry Sciences, Huazhong Agricultural University, Wuhan 430070, China; Hubei Engineering Technology Research Center for Forestry Information, Wuhan 430070, China; College of Horticulture & Forestry Sciences, Huazhong Agricultural University, Wuhan 430070, China; National Key Laboratory for Germplasm Innovation & Utilization of Horticultural Crops, Wuhan 430070, China; College of Horticulture & Forestry Sciences, Huazhong Agricultural University, Wuhan 430070, China; Hubei Engineering Technology Research Center for Forestry Information, Wuhan 430070, China

## Abstract

Analyzing and comparing the effects of labor-saving cultivation modes on photosynthesis, as well as studying their vertical canopy architecture, can improve the tree structure of high-quality and high-yield citrus and selection of labor-saving cultivation modes. The photosynthesis of 1080 leaves of two labor-saving cultivation modes (wide-row and narrow-plant mode and fenced mode) comparing with the traditional mode were measured, and nitrogen content of all leaves and photosynthetic nitrogen use efficiency (PNUE) were determined. Unmanned aerial vehicle (UAV)-based light detection and ranging (LiDAR) data were used to assess the vertical architecture of three citrus cultivation modes. Results showed that for the wide-row and narrow-plant and traditional modes leaf photosynthetic CO_2_ assimilation rate, stomatal conductance, and transpiration rate of the upper layer were significantly higher than those of the middle layer, and values of the middle layer were markedly higher than those of the lower layer. In the fenced mode, a significant difference in photosynthetic factors between the upper and middle layers was not observed. A vertical canopy distribution had a more significant effect on PNUE in the traditional mode. Leaves in the fenced mode had distinct photosynthetic advantages and higher PNUE. UAV-based LiDAR data effectively revealed the differences in the vertical canopy architecture of citrus trees by enabling calculating the density and height percentile of the LiDAR point cloud. The point cloud densities of three cultivation modes were significantly different for all LiDAR density slices, especially at higher canopy heights. The labor-saving modes, particularly the fenced mode, had significantly higher height percentile data.

## Introduction

Photosynthesis is the most important chemical reaction on Earth, providing the oxygen, food, and energy needed for human survival. The continuous improvement of the photosynthetic efficiency of different crops is markedly correlated with an increase in yield [1–3]. Improving photosynthetic efficiency is one of the most important ways to increase productivity, with further increases in yield potential largely depending on improved photosynthesis [4, 5]. Photosynthesis is a key factor that restricts crop yields because yield is closely related to light energy received per unit area of land, with factors including the interception efficiency of light energy by the plant canopy, the efficiency with which plants convert light energy into chemical energy and store it in biomass, and the harvest index [4, 6]. Canopy architecture is the main factor that determines yield because it affects light distribution, light interception, and the corresponding canopy photosynthesis [7]. The most general strategy for improving conversion efficiency (${\varepsilon}_c$) at the canopy level involves changing the canopy architecture and size to improve the light distribution of dense-canopy crops [4], maximize nitrogen distribution, and increase photosynthetic rate [8]. The productivity of fruit trees is also roughly proportional to light interception, although the relationship is weaker when >50% of the available light is intercepted [9–11].

Citrus is the world’s largest type of fruit, and it is cultivated in the widest area of southern China. New modes of labor-saving cultivation focus on changing density to thinning, wide rows and dense plants, ridge cultivation, water and fertilizer integration, and mechanical operations. This development of new modes and technologies in the area of citrus production has increased yearly [12]. The main features of this type of cultivation include small plant spacing and large row spacing compared with the traditional mode, which has a smaller row spacing and larger plant spacing without pruning, which limits mechanized cultivation management and robotic picking. Two innovative and labor-saving cultivation modes, namely the wide-row narrow-plant mode and the fenced mode, which are suitable for apples, pears, apricots, cherries, and other tree species, have also been applied in the cultivation of citrus trees. Compared with methods used for other fruit trees, these new labor-saving cultivation modes have the advantages of enabling the management of shoots at the adult stage and reducing losses caused by Huanglong disease, in addition to improved ventilation and light transmission. The canopy of the fenced mode is maintained relatively small by regular pruning. The fenced pattern has half the plant spacing of the wide-row narrow-plant pattern, which leads to the setting of a hedge frame in the row for fixing citrus trees [13]. Pruning and planting techniques cause large differences in canopy structure among the three modes. The cultivation pattern has a significant impact on fruit quality, mainly in the quality of a single fruit, peel thickness, soluble solids, solid acid ratio, juice yield, and Vitamin C content [14]. Fruits obtained using the wide-row and narrow-plant mode and the fenced mode have higher soluble solids, juice yield, solid acid ratio, and Vc content. Interestingly, the canopy layer does not have a significant impact on the fruit quality of the fenced pattern but has a greater impact on the wide-row and narrow-plant patterns and traditional cultivation patterns [14]. These findings may contribute to the optimization of a potential strategy for optical interception and the effective improvement of light distribution within the canopy. Engineering, such as altering the cultivation mode for more efficient light capture and utilization, provides a sustainable strategy for increasing crop yields [15]. Canopy architectures may also mitigate light saturation of the upper canopy and allow more light to reach the lower leaves, thereby improving photosynthetic efficiency.

Light detection and ranging (LiDAR) is an active sensing technology that uses the laser speed and flight time recorded by a timer to send a short-wavelength laser to measure the distance between the sensor and target [16]. LiDAR has been widely used in the quantitative analysis of plant structures, and it can penetrate the vegetation canopy to characterize the internal structure of vegetation [17–23]. Advances in the use of LiDAR technology to obtain horticultural structural information has ranged from the canopy to the organ level and can be divided into geometric characterization and organ detection [24]. There are also many applications of LiDAR in fruit trees, such as vineyards, apples [25], oranges [23], and olives [26]. However, most use terrestrial laser scanner modeling to obtain geometric canopy information, including volume and height. At present, few applications have used LiDAR technology to compare the canopy characteristics of different fruit tree cultivation pattern groups.

How does the cultivation mode affect the photosynthetic characteristics of citrus trees? What are the advantages of the new labor-saving cultivation methods in terms of improving the photosynthetic efficiency of citrus trees? What are the differences in the internal vertical structures of citrus tree groups trained using different cultivation modes? To answer these questions, this study focused on two new cultivation modes with different potentials for canopy light interception and distribution for two purposes: (i) from the perspective of population, we revealed the differences in photosynthetic factors and photosynthetic nitrogen use efficiency (PNUE) of citrus trees under different cultivation methods, and present the differences between different canopy levels; and (ii) we used unmanned aerial vehicle (UAV)-based LiDAR technology to explain the mechanism of cultivation modes and hierarchical structure, which causes differences in photosynthesis and photosynthetic nitrogen use efficiency.

## Results

### Variation in leaf photosynthesis and nitrogen photosynthetic utilization by different layers

For the wide-row and narrow-plant mode and the traditional mode, the leaf *Pn* values of the upper layer were significantly larger than those of the middle layer. The *Pn* values of the middle layer were significantly higher than those of the lower layers. Leaf *Cond* and *Trmmol* showed similar trends (Fig. 2A, C, D, F, G, and I). For the fenced mode, the leaf *Pn* values of the upper layer were not higher than those of the middle layer; the *Pn* difference between the middle and lower layers was also not significant; and the *Pn* values of the upper layer were significantly larger than those of the lower layer (Fig. 2B). The leaf *Trmmol* values of the upper and middle layers were significantly higher than those of the lower layer when citrus trees were cultivated in the fenced mode (Fig. 2H).

When citrus trees were cultivated in the wide-row and narrow-plant mode, the PNUE values of the upper and middle layers were obviously higher than those of the lower layer; those of the upper and middle layers did not differ significantly (Supplementary Data Fig. S1A). For the trees in fenced mode, the PNUE values of the upper layer were higher than those of the middle layer; those of the upper and lower layers were not significantly different, and the lower leaves showed strong photosynthetic nitrogen use ability (Fig. S1B). For the citrus trees cultivated in traditional mode, the level had a very significant impact on PNUE; the PNUE values of the upper layer were significantly higher than those of the middle layer, and those of the middle layer were markedly higher than those of the lower layer (Supplementary Data Fig. S1C).

### Effect of cultivation mode on leaf photosynthesis, nitrogen photosynthetic utilization, and effective leaf area index (LAIe)

The fenced mode was found to increase the photosynthetic rate. Regardless of the location of the leaves, whether in the upper, middle, or lower layer, the leaf *Pn* values in fenced mode were greater than those of the wide-row and narrow-plant or traditional mode, and the values of the wide-row and narrow-plant mode and the traditional mode were not significantly different (Fig. 3A–C). For the leaves in the upper and lower layers, the stomatal conductance of the leaves in fenced mode was larger than that in traditional mode. The *Cond* values of the traditional mode were significantly larger than those of the wide-row and narrow-plant mode (Fig. 3D and F). The highest values of *Cond* were observed in the leaves of the middle layers cultivated in traditional mode (Fig. 3E). Transpiration was also affected by the cultivation mode. The leaves of the wide-row and narrow-plant mode had the lowest *Trmmol* values (Fig. 3G–I). The PNUE values of leaves in the upper and middle layers of the fenced and traditional modes were higher than those of the wide-row and narrow-plant mode. The PNUE value of leaves in the lower layer of the fenced mode was the highest, and no significant difference was observed between the traditional mode and the wide-row and narrow-plant mode (Supplementary Data Fig. S2). The mean LAIe value of the leaves in fenced mode was significantly larger than that of the leaves in the wide-row and narrow-plant mode and the traditional mode (Supplementary Data Fig. S3).

### Canopy vertical distribution characteristics of different cultivation modes

Figure 4 shows the point cloud density of each citrus tree in the wide-row and narrow-plant, fenced, and traditional modes for 10 low- to high-density slices. The point-cloud densities of the three cultivation modes were significantly different for all density slices (Fig. 4). The point cloud density of the wide-row and narrow-plant mode and the fenced mode at higher height slices (i.e.. *d*_*6*_ to *d*_*10*_) was significantly higher than that of the traditional mode (Table 1). The point cloud density for more than half of the tree height was 64.85% in the wide-row and narrow-plant modes, 71.94% in the fenced mode, and 50.02% in the traditional mode Table 1. The density slices where the point cloud density of the fenced mode was >10% were from* d_5_*-*d_9_*, those with wide-row and narrow-plant mode were from *d_5_*-*d_8_*, and the traditional mode was from* d*_*4*_-*d*_*8*_. Interestingly, the fenced mode achieved 14.52% accuracy on high height-density slices* d*_*9*_ (Table 1).

**Table 1:** Mean density values (%) of the point cloud in the wide-row and narrow-plant, fenced, and traditional cultivation modes. *d*_1_, *d*_2_, *d*_3_, *d*_4_, *d*_5_, *d*_6_, *d*_7_, *d*_8_, *d*_9_, and *d*_10_ are low- to high-density slices

Cultivation pattern	*d*_1_	*d*_2_	*d*_3_	*d*_4_	*d*_5_	*d*_6_	*d*_7_	*d*_8_	*d*_9_	*d*_10_	Sum
Wide-row and narrow-plant	2.69	3.55	5.51	9.13	14.26	18.54	19.75	15.92	8.44	2.20	100
Fenced	3.03	3.68	5.27	6.95	10.13	12.16	19.70	20.52	14.52	4.03	100
Traditional	6.22	7.00	9.21	12.83	14.72	16.04	15.08	11.64	5.74	1.52	100

The height percentiles with or without ground point data for each citrus tree in the three cultivation modes are shown in Figs 5 and 6. The *h_p_* and *h_gp_* of the different cultivation modes presented similar trends. The 15 height percentiles of the fenced mode were significantly larger than those of the wide-row narrow-plant mode, and the parameters of the wide-row narrow-plant mode were significantly larger than those of the traditional mode (Figs 5 and 6). The mean values of the 99% height percentile of the wide-row and narrow-plant, fenced, and traditional cultivation modes were 2.11, 2.83 , and 1.94 m, respectively (Table 2). The mean heights of the 50% point clouds without ground points of the wide-row and narrow-plant, fenced, and traditional cultivation modes were 1.43, 2.04, and 1.19 m, respectively (Table 3).

**Table 2:** Mean height percentile values with ground points in the wide-row and narrow-plant, fenced, and traditional cultivation modes. 1%, 5%, 10%, 20%, 25%, 30%, 40%, 50%, 60%, 70%,75%, 80%, 90%, 95%, and 99% indicate the percentage of the point cloud density data (m)

Cultivation pattern	*h*_g1%_	*h*_g5%_	*h*_g10%_	*h*_g20%_	*h*_g25%_	*h*_g30%_	*h*_g40%_	*h*_g50%_	*h*_g60_%	*h*_g70%_	*h*_g75%_	*h*_g80%_	*h*_g90%_	*h*_g95%_	*h*_g99%_
Wide-row and narrow-plant	0.40	0.69	0.86	1.07	1.15	1.22	1.33	1.43	1.52	1.62	1.66	1.72	1.85	1.94	2.09
Fenced	0.43	0.78	1.05	1.42	1.56	1.68	1.88	2.04	2.17	2.29	2.35	2.41	2.54	2.64	2.80
Traditional	0.31	0.45	0.60	0.82	0.90	0.97	1.08	1.19	1.28	1.38	1.44	1.50	1.64	1.74	1.90

**Table 3:** Mean height percentile values without ground points in the wide-row and narrow-plant, fenced, and traditional cultivation modes. 1%, 5%, 10%, 20%, 25%, 30%, 40%, 50%, 60%, 70%,75%, 80%, 90%, 95%, and 99% indicate the percentage of the point cloud density data (m)

Cultivation pattern	*h*_1%_	*h*_5%_	*h*_10%_	*h*_20%_	*h*_25%_	*h*_30%_	*h*_40%_	*h*_50%_	*h*_60%_	*h*_70%_	*h*_75%_	*h*_80%_	*h*_90%_	*h*_95%_	*h*_99%_
Wide-row and narrow-plant	0.58	0.89	1.04	1.22	1.29	1.34	1.44	1.53	1.61	1.69	1.74	1.79	1.90	1.98	2.11
Fenced	0.69	1.14	1.42	1.73	1.84	1.92	2.07	2.18	2.28	2.38	2.43	2.48	2.60	2.69	2.83
Traditional	0.41	0.67	0.83	1.00	1.07	1.12	1.22	1.31	1.40	1.49	1.54	1.59	1.71	1.80	1.94

Supplementary Data Fig. S4 presents the LiDAR point cloud vertical distribution and the corresponding fitting curves of the different cultivation modes. The point cloud distribution curve of the traditional mode showed a spiky shape with increasing height, and was more concentrated. The vertical point cloud distribution of the fenced and wide-row narrow-plant modes was relatively discrete. The maximum point density of LiDAR in the fenced mode was significantly higher than that in the wide-row narrow-plant and traditional modes.

## Discussion

In this study, we compared the photosynthesis and PNUE differences in the canopy layers of citrus trees under different cultivation modes by conducting extensive survey work. These differences were then analyzed using UAV-based LiDAR point cloud data of the different cultivation modes to elucidate the mechanisms underlying the labor-saving cultivation modes to gain insights into the improved photosynthetic capacity and canopy structure of citrus trees.

**Figure 1:**
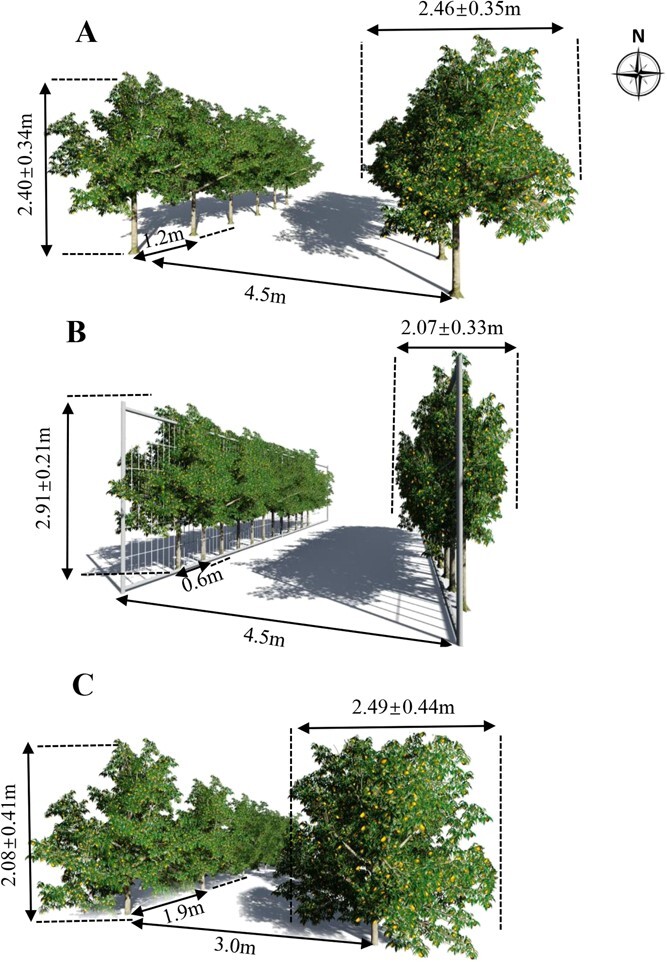
Summary of inter-row plant spacing, within-row tree spacing, crown width, tree height, and diameter under three cultivation patterns. Crown width, tree height, and diameter are presented as the mean ± standard deviation. Three cultivation modes are presented: (A) wide-row and narrow-plant mode; (B) fenced mode; and (C) traditional mode.

**Figure 2:**
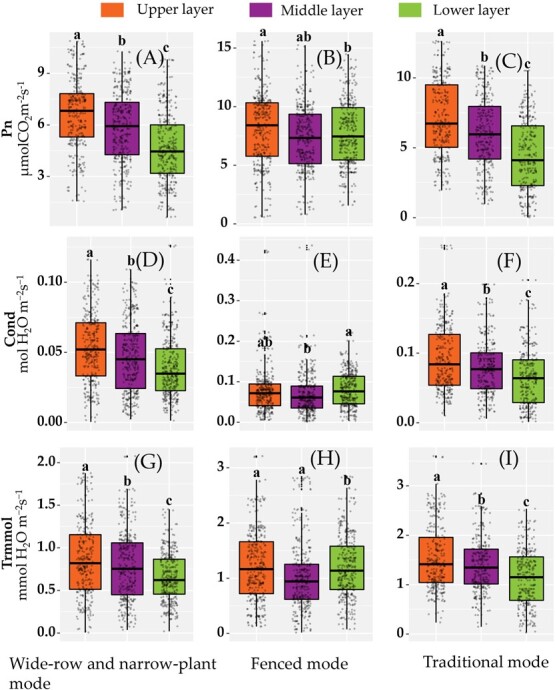
One-way ANOVA results for photosynthetic rate (*Pn*, μmolCO_2_ m^−2^ s^−1^) of the different cultivation modes. A, D, and G represent *Pn*, *Cond*, and *Trmmol*, respectively, of the wide-row and narrow-plant modes; B, E, and H represent *Pn*, *Cond*, and *Trmmol*, respectively, of the fenced mode; C, F, and I represent *Pn*, *Cond*, and *Trmmol*, respectively, of the traditional mode.

Training and pruning techniques in fruit trees can result in variability in canopy architecture, which affects the photosynthetic capacity of leaves [27, 28]. In this study, we found that the canopy layer significantly affected the photosynthetic capacity and PNUE of citrus leaves, especially for citrus trees cultivated in the traditional mode and the wide-row narrow-plant mode; however, this effect was not as marked in the fenced mode (Fig. 2). The absorption of red and blue light by chlorophyll a and b in plant chloroplasts leads to a decline in light intensity with canopy depth [15]. This phenomenon was more evident in the traditional citrus cultivation mode because of the smaller row spacing (Fig. 1). The new labor-saving cultivation modes, especially the fenced mode, can effectively reduce the interception and shading of light between the leaves, with a wide row spacing and narrow crown width due to trimming [13]. Previous studies have shown that hedgerows can improve the canopy light distribution of mainly apple trees mainly [29]. However, little attention has been paid to citrus trees.

**Figure 3:**
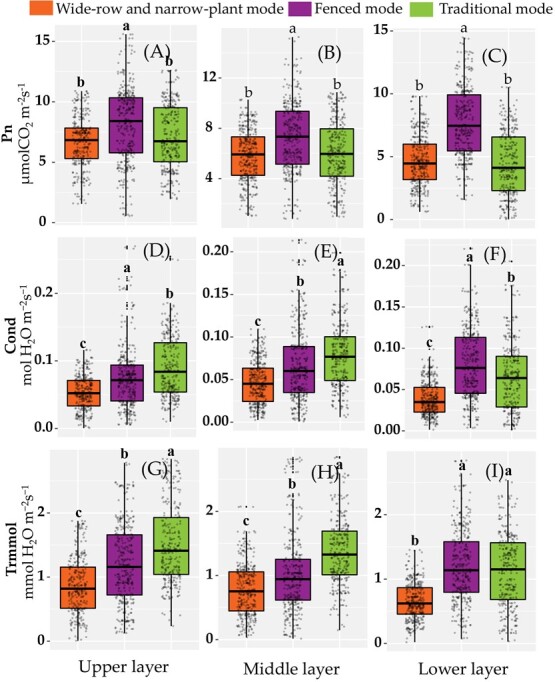
*Pn* (μmolCO_2_ m^−2^ s^−1^) values in wide-row and narrow-plant mode, fenced mode, and traditional mode. (A, D, G) Upper layer; (B, E, H) middle layer; (C, F, I) lower layer.

In the present study, we proved that the cultivation mode can significantly affect photosynthetic rate and PNUE in citrus trees. As a new labor-saving cultivation mode, the fenced mode increased the photosynthetic rate regardless of whether the leaves were located in the upper, middle, or lower layers. The photosynthetic rate of the citrus leaves in the fenced mode was significantly higher than that in the wide-row and narrow-plant mode or the traditional mode (Fig. 3). This advantage was more pronounced in the lower leaves. The mean *Pn* values of the lower leaves in fenced mode were 1.74 times that in traditional mode and 1.66 times that in wide-row and narrow-plant mode. These results may contribute to potential strategies to improve light distribution and light use efficiency in citrus tree canopies using fenced mode. Wide row spacing and unique pruning strategies resulted in a low density and distribution of leaves inside citrus trees [13]. Light interception affects the photosynthetic capacity of leaves [30]. Since the canopies of citrus trees in the fenced mode had the highest LAI, they were able to optimize more light interception over a larger area (Supplementary Data Fig. S3).

**Figure 4:**
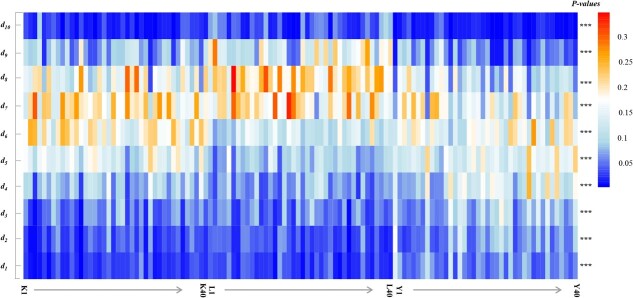
Heat map representing the density of point cloud data in the different cultivation patterns (K is wide-row and narrow-plant mode, L is fenced mode, and Y is traditional mode). The *x*-axis indicates the different cultivation patterns; each cultivation pattern included 40 individuals. The *y*-axis indicates the density of point cloud data; *d*_1_, *d*_2_, *d*_3_, *d*_4_, *d*_5_, *d*_6_, *d*_7_, *d*_8_, *d*_9_, and *d*_10_ are low- to high-density slices. One-way ANOVA was performed for the three cultivation patterns. ^***^P < .001.

To elucidate the mechanisms underlying the effects of the citrus canopy architecture on the *Pn* and PNUE values in different cultivation modes, we analyzed the internal vertical distribution characteristics of UAV-based LiDAR point cloud data. Significant differences were observed in the vertical density distribution of the point clouds among the three cultivation modes, especially at higher canopy heights (Fig. 4). For the point cloud density of more than half the tree height, the fenced mode reached 71.94% and the traditional mode was the lowest, at 50.02% (Table 1). The 99 and 50% height percentiles without ground points showed that the fence mode was significantly higher than the wide-row and narrow-plant mode, and that the wide-row and narrow-plant mode was higher than the traditional mode (Fig. 6 and Table 3). These results indicate that the new cultivation modes, including more leaves that were distributed in the middle and upper layers, could intercept more light for photosynthesis. Therefore, the lower leaves of the fenced mode could avoid shading, and the photosynthetic rate of lower leaves was not significantly different from that of middle leaves, while the photosynthetic rate of lower leaves in the traditional mode and the wide-row and narrow-plant mode was significantly inhibited (Fig. 2). Therefore, improving light distribution by changing the canopy architecture via the cultivation mode is associated with greater photosynthesis and photosynthetic nitrogen utilization in citrus trees.

## Conclusions

Labor-saving cultivation modes, such as the fenced mode and the wide-row and narrow-plant mode, have been gradually applied within the cultivation of citrus trees. These new cultivation modes that modify canopy architecture have shown success in improving light efficiency, thereby significantly increasing photosynthetic rate and PNUE. Regardless of the location of the leaves in the canopy, the leaf *Pn* values of the leaves of trees in the fenced mode were higher than in the wide-row and narrow-plant mode or traditional mode. The canopy layers had a more significant effect on *Pn*, *Cond*, *Trmmol*, and PNUE than in the traditional mode or wide-row and narrow-plant mode. UAV-based LiDAR effectively revealed the mechanisms underlying the differences in photosynthesis in citrus trees under three cultivation modes using the mean density, height percentile, cumulative height percentile, and fitting canopy distribution curves.

## Materials and methods

### Plant material and experimental design

This study was carried out from 15 November to 1 December in 2020 in a 5-year-old healthy Newhall Navel Orange orchard, where rows with zero azimuth were planted on the plain in the southern area of Jiangxi Province. The three cultivation methods applied were the wide-row and narrow-plant, fenced, and traditional modes. Different tree-crown shapes imposed by tree pruning and different within-row tree spacing and plant spacing characterized the three selected cultivation modes (Fig. 1). The wide-row and narrow-plant mode had a row width of 4.5 m, a plant spacing of 1.2 m, an average crown width of 2.46 m, and a tree height of 2.4 m. The fenced mode had a row width of 4.5 m, plant spacing of 0.6 m, an average crown width of 2.07 m, and a tree height of 2.91 m. In comparison, the traditional mode had a row width of 3.0 m, a plant spacing of 1.9 m, an average crown width of 2.49 m, and a tree height of 2.08 m. A total of 120 citrus trees (40 in each mode) were selected for the investigation. Each tree was positioned using differential GPS with an error of 8 mm. All trees were grown with adequate water and nutritional supplies, irrigation, and fertilization schemes, applied to citrus trees of the three cultivation modes throughout the growing season. The inter-row plant spacing, within-row tree spacing, crown width, tree height, and diameter under the three cultivation patterns are shown in Fig. 1. The photosynthetic factors and nitrogen content of nine leaves from the upper, middle, and lower layers of each citrus tree were measured. The upper, middle, and lower layers were determined based on canopy height. The top third of the canopy height was defined as the upper layer, the middle third was defined as the middle layer, and the lower third was defined as the lower layer. Each layer of selected leaves comprised four to six leaves from the tip of the spring shoots of the annual branches. A total of 1080 leaves were used in this study.

### Photosynthetic measurement

Each selected leaf was placed into the blade chamber of a LICOR 6400XT gas analyzer (LICOR Biosciences, Lincoln, NE, USA) with an attached red–blue light leaf chamber under saturated light (1000 mmol m^−2^ s^−1^), a flow rate of 500 mmols^−1^, and a block temperature of 25°C. We measured the leaf photosynthetic CO_2_ assimilation rate (*Pn*, μmol CO_2_ m^−2^ s^−1^), leaf stomatal conductance (*Cond*, mol H_2_O m^−2^ s^−1^), and leaf transpiration rate (*Trmmol*, mmol H_2_O m^−2^ s^−1^) for each leaf after an adjustment period of ~30 min. The measurement time was controlled between 9:00 a.m. and 11:00 a.m.

**Figure 5:**
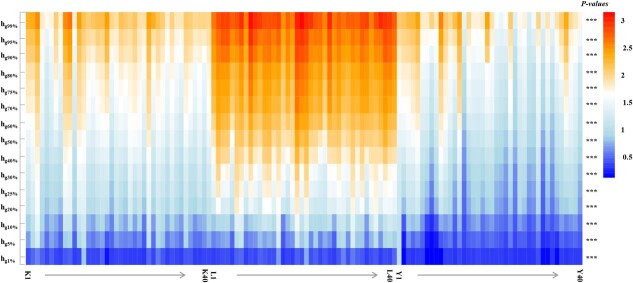
Heat map representing the height percentile with ground points in the different cultivation patterns (K is wide-row and narrow-plant mode, L is fenced mode, and Y is traditional mode). The *x*-axis indicates the different cultivation patterns; each cultivation pattern included 40 individuals. The *y*-axis indicates the height percentile with ground points of 1, 5, 10, 20, 25, 30, 40, 50, 60, 70,75, 80, 90, 95, and 99%. One-way ANOVA was performed among the three cultivation patterns. ^***^P < .001.

**Figure 6:**
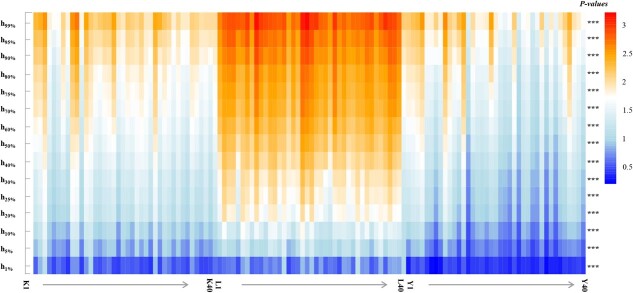
Heat map representing the height percentile without ground points in the different cultivation patterns (K is wide-row and narrow-plant mode, L is fenced mode, and Y is traditional mode). The *x*-axis indicates the different cultivation patterns; each cultivation pattern included 40 individuals. The *y*-axis indicates the height percentile without ground points of 1, 5, 10, 20, 25, 30, 40, 50, 60, 70,75, 80, 90, 95, and 99%. One-way ANOVA was performed among the three cultivation patterns. ^***^P < .001.

### Determination of N concentrations

The total N concentration was analyzed using the Kjeldahl method, as reported by Li *et al*. [31]. The N amounts in the leaves were calculated from N concentrations and biomass, according to Li *et al*. [32].

### Determination of photosynthetic nitrogen use efficiency

PNUE was calculated as follows:

(1) \begin{align*} PNUE=Pn/N \end{align*}

where *Pn* is leaf photosynthetic CO_2_ assimilation rate and *N* is leaf total N concentration.

### Determination of effective leaf area index

WinSCANOPY 2020 and the supporting software (Canada) were used to estimate the effective leaf area index (LAIe) of 120 citrus trees before sunrise or after sunset.

### LiDAR data processing and variable extraction

Laser point cloud data were obtained using a laser scanner carried by an unmanned aerial vehicle (UAV) (Dajiang M600; China). The average point cloud density was ~360 points m^−2^. Laser point data were first classified and a digital elevation model (DEM) was generated using the triangular irregular network (TIN) interpolation method. Then, the height of the vegetation echo point was normalized by the elevation value of the DEM, and the elevation of the terrain was removed, which can generate the normalized vegetation LiDAR points. The metrics canopy height at different percentiles and density at different canopy heights were derived from normalized vegetation LiDAR points, which are widely used to characterize the vertical structure of vegetation [33, 34]. Percentiles can accurately reflect the distribution of the laser point data in the vertical direction [35]. The height percentile refers to sorting all normalized LiDAR point clouds by height in a statistical unit. The height p% of the points in each statistical unit was calculated using Equation (2):

(2) \begin{equation*} P\left(h\le{h}_p\right)=p \end{equation*}

where *P*() represents the normalized probability of height *h*, *p* represents the percentile of the LiDAR points, and *h_p_* is the height at percentile *p*. Fifteen height percentiles were extracted: *h*_1%,_*h*_5%_, *h*_10%_, *h*_20%_, *h*_25%_, *h*_30%_, *h*_40%_, *h*_50%_, *h*_60%_, *h*_70%_, *h*_75%_, *h*_80%_, *h*_90%_, *h*_95%_, and *h*_99%_. The 99.9th percentile of the points was defined as plant height, which was represented using the variable *h*. In this study, we calculated two types of height percentile variable: the first removed the ground points and was represented by *h_p_*, and the second did not remove the ground points and was represented by *h_gp_*.

The density at different canopy heights was calculated using Equation (3):

(3) \begin{equation*} {d}_i={\sum}_{\left(i-1\right)\frac{Z_{\max -{Z}_{\mathrm{min}}}}{10}}^{i\frac{Z_{\max -{Z}_{\mathrm{min}}}}{10}}{n}_i/N \end{equation*}

where *d_i_* is the cumulative density at layer height *i* (*i* = 1–10), *N* is the total number of LiDAR points, *n_i_* is the number of LiDAR points at layer *i*, and *z*_min_ and *z*_max_ represent the lowest and highest values of the *z* value in LiDAR points. Ten density variables, *d*_1_ to *d*_10_, were also extracted.

## Acknowledgements

This work was supported by the National Key Research and Development Plan (no. 2019YFD1000104) and a project supported by the Fundamental Research Funds for the Central University (no. 2662020YLPY020). This study was also supported by two National Natural Fund Projects (no. 31901963 and 31972356) and an earmarked fund for CARS 26.

## Author contributions

Y.-Y.D. obtained LiDAR data using a UAV and extracted and analyzed the corresponding parameters of the point cloud data. X.-Y.L., L.-H., J.-X.Z., and W.-B.Z. measured all photosynthetic and nitrogen contents. J.-Z.Z. and C.-G.H. found appropriate citrus orchards and helped determine the experimental design. Y.-Z.L. checked the experimental data and revised the manuscript accordingly. Q.-Q.H and Y.-H.Z helped to determinte leaf nitrogen cotents and draw figures. Y.-N.F. coordinated the laboratory instruments and assisted with nitrogen determination. J.-J.Z. designed the experiment, analyzed the photosynthetic factors and nitrogen content-related experimental data, and wrote the manuscript. All the authors have read and agreed to the published version of the manuscript.

## Data availability

The data used in this study are available from the corresponding author upon reasonable request.

## Conflict of interest

The authors declare no conflict of interest.

## Supplementary Data

Supplementary data is available at *Horticulture Research* online.

## Supplementary Material

Web_Material_uhad018
