# Supplementary material for: Characteristics of photosynthesis and vertical canopy architecture of citrus trees under two labor-saving cultivation modes using unmanned aerial vehicle (UAV)-based LiDAR data in citrus orchards
Source: Hortic Res. 2023 Feb 8;10(3):uhad018. doi: 10.1093/hr/uhad018 (PMC10031737; doi:10.1093/hr/uhad018)
Supplement: Web_Material_uhad018 [file web_material_uhad018.zip › æ-°å»o Microsoft Word æ-╪æ¡£.pdf]

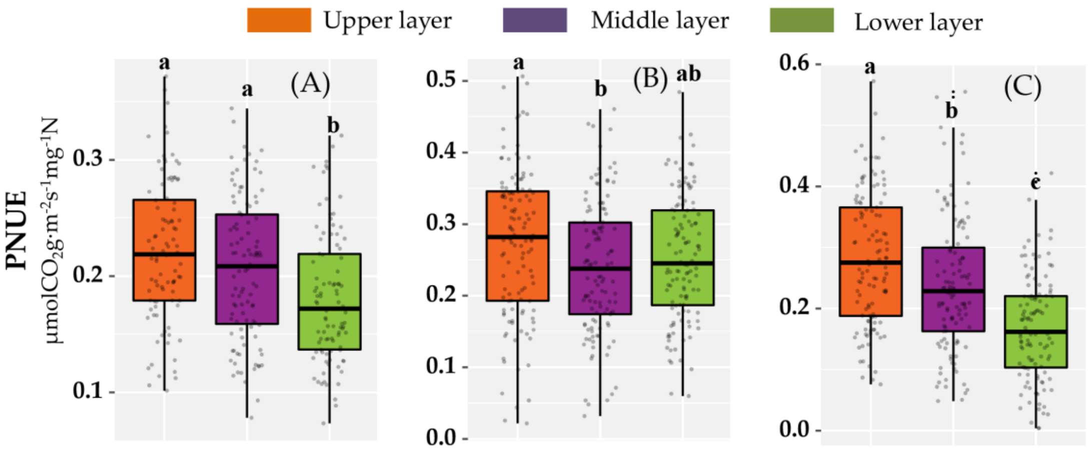

Figure S1: **One-way ANOVA results for photosynthetic nitrogen use efficiency (PNUE, μmolCO_2m^{-2}s^{-1}mg^{-1}N) in different cultivation modes.** (A) Wide-row and narrow-plant mode; (B) fenced mode; (C) traditional mode.

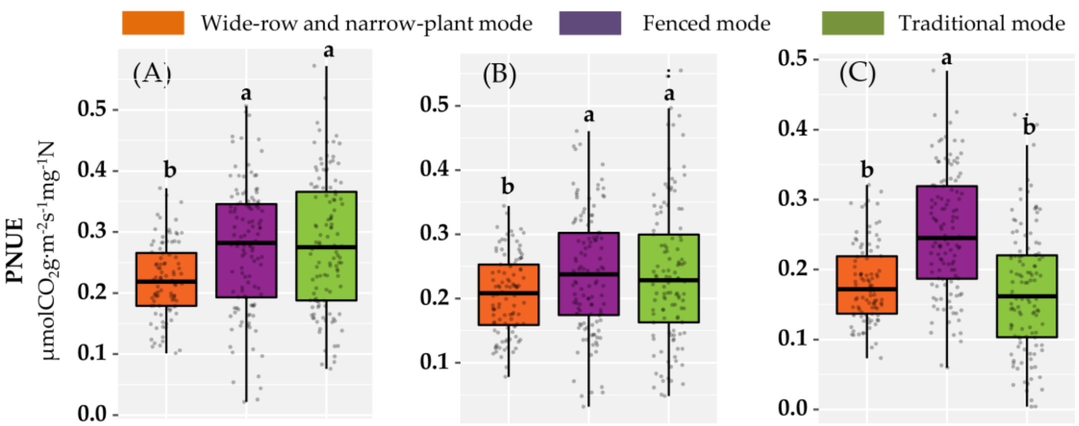

Figure S2: **Statistical analysis of photosynthetic nitrogen use efficiency in leaves. (**A) upper layer; (B) middle layer; (C) lower layer.

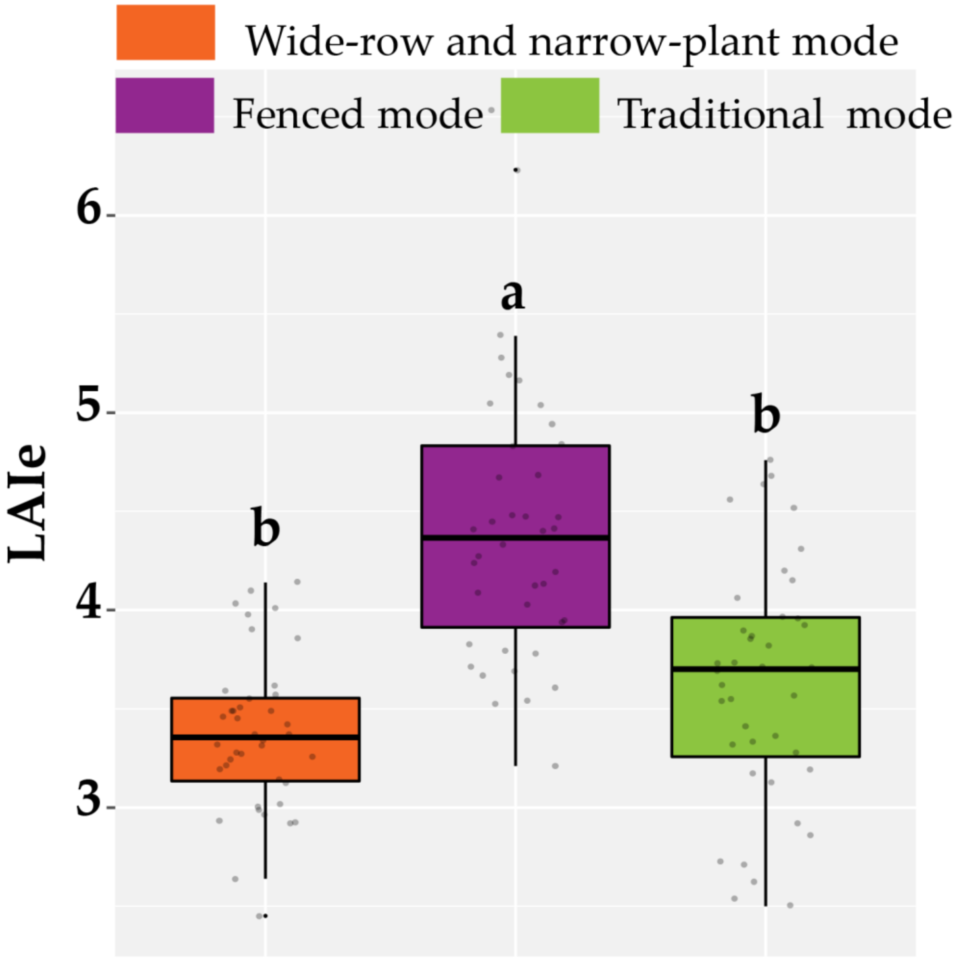

Figure S3: **Performance of LAIe values of wide-row and narrow-plant mode, fenced mode, and traditional mode.**

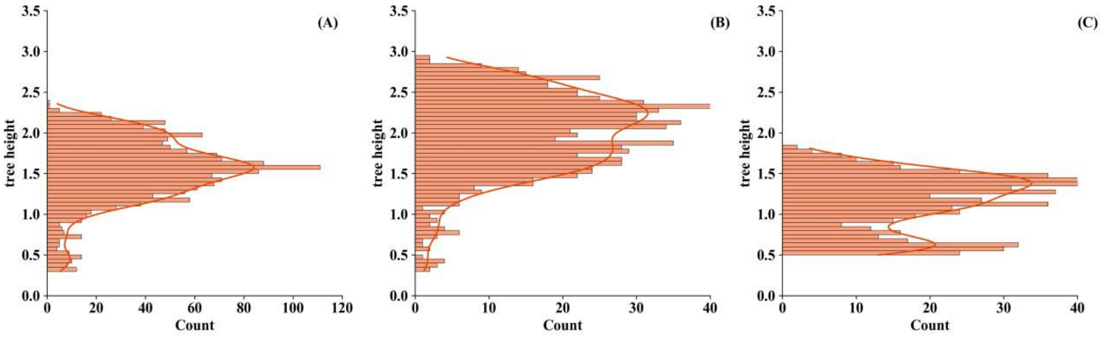

Figure S4: **Canopy distribution and fitting curves of three cultivation modes.** (A) Wide-row and narrow-plant mode; (B) fenced mode; (C) traditional mode.
